# A Chinese patient with *POLR3A*-related leukodystrophy: a case report and literature review

**DOI:** 10.3389/fneur.2023.1269237

**Published:** 2023-10-27

**Authors:** Lei Sun, Weihong Lin, Hongmei Meng, Wuqiong Zhang, Shuai Hou

**Affiliations:** Department of Neurology, The First Hospital of Jilin University, Changchun, China

**Keywords:** *POLR3A*, hypomyelinating leukodystrophy-7, 4H syndrome, diagnosis, demyelinating diseases

## Abstract

**Background:**

Leukodystrophies are hereditary white matter diseases characterized by genetic polymorphisms and considerable phenotypic variability. They can be classified into myelin and non-myelin malformations. These diseases are rare, affecting 1 out of 250,000–500,000 individuals and can manifest at any age. A subtype of leukodystrophy, associated with missense mutations in the RNA polymerase subunit III (*POLR3A*) gene, is inherited in an autosomal recessive manner.

**Case report:**

We report and analyse a case of a 34-year-old female who presented with ataxia. Magnetic Resonance Imaging (MRI) of the brain revealed demyelinating lesions in the white matter. Genetic testing identified the c.4044C > G and c.1186-2A > G variants in the *POLR3A* gene. The patient was diagnosed with hypomyelinating leukodystrophy type 7 and received neurotrophic and symptomatic supportive therapy. However, after 1 month of follow-up, there was no improvement in her symptoms.

**Conclusion:**

*POLR3A*-induced leukodystrophy is relatively rare and not well understood, making it challenging to diagnose and easy to overlook. The prognosis for this disease is generally poor, significantly impacting the quality of life of affected individuals. Currently, no cure is available for this condition, and treatment is limited to managing symptoms. Further research into new treatment methods for *POLR3A*-induced leukodystrophy is imperative to improve the quality of life and potentially extend the life expectancy of patients.

## 1. Introduction

Leukodystrophy is a group of rare hereditary diseases with multiple inheritance patterns, including X-linked, autosomal dominant, and autosomal recessive inheritance patterns, and may also be caused by pathogenic variants of hundreds of genes encoding proteins involved in RNA translation, such as RNA polymerase three subunit A (*POLR3A*), *POLR3B, POLR1C*, and *POLR3K* (1). It is characterized by white matter abnormalities in the central nervous system (CNS) and often leads to progressive neurodegeneration and premature death (2).

Depending on whether white matter abnormalities observed on brain magnetic resonance imaging (MRI) result from insufficient initial myelin deposition or alterations in myelin balance, leukodystrophy can be classified as either poorly myelinated or non-myelinated (3). Hypomyelinating leukodystrophy type 7 (HLD 7) is an autosomal recessive oligodendrocyte-associated myelin disorder associated with several nucleotide mutations in the *POLR3A* gene (4). Because such diseases are rare, we report a case of leukodystrophy caused by a missense mutation in *POLR3A* and review the relevant literature to integrate diagnostic experience and provide ideas for the diagnosis of such diseases in the future.

## 2. Case report

A 34-year-old Han female patient with ataxia was admitted to our department. The patient had a slight limp without obvious inducement 7 or 8 years previously. Approximately 1 year prior to the presentation, she had given birth to a boy and subsequently developed progressive walking instability. Five months prior to the evaluation, she started experiencing static tremors in her hands and head, difficulty speaking, occasional numbness in the limbs, and a cough after drinking water. The tremor was significantly aggravated during movement. The patient also reported headaches, dizziness, and nausea. One year prior, she lacked appetite and had lost more than 10 pounds. The patient had a history of cephalosporin allergy and had undergone an appendectomy 20 years prior. She had a history of tobacco and alcohol use for 10 years and had quit smoking 6 months ago. Her parents were still alive, and she was the only child. She also reported that she had no relatives with similar symptoms. She had 27 teeth, a smaller tooth size, and normal sex hormone levels. And her menstruation was normal. At the time of presentation, she had dysarthria and upbeating nystagmus, with right gaze-evoked nystagmus. Her limb reflexes were absent, and her bilateral finger-nose test was unstable. She demonstrated instability on her calcaneus tibial test, and had a positive Romberg sign and bilateral Babinski and Chaddock's signs.

**Figure 1:**
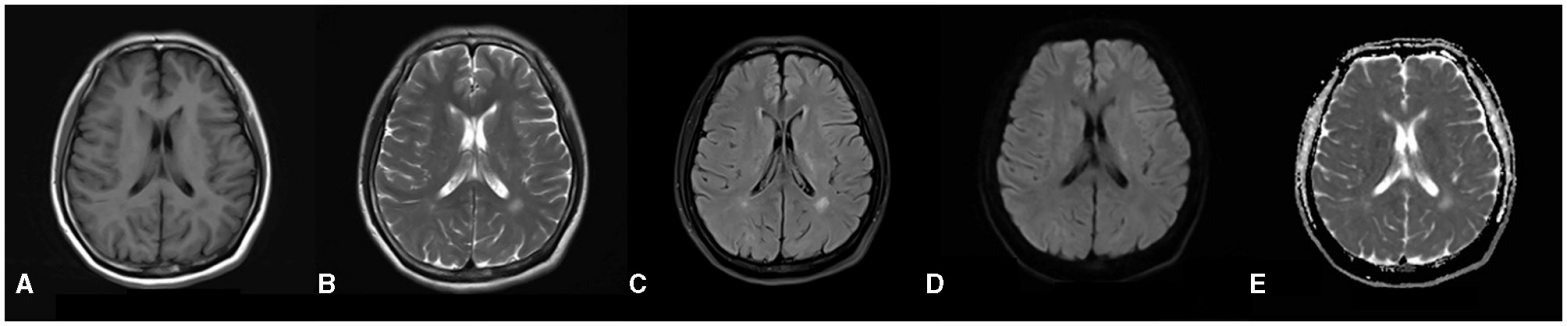
**(A–E)** Brain MRI reveals a distribution of abnormal signals localized around the ventricles, as well as within the frontal and temporal lobes. In the T1-weighted images, there is a depiction of slightly hypo-intense signals **(A)**, while the T2-weighted images illustrate hyper-intense signals **(B)**. The dark-field images similarly exhibit hyper-intense signals **(C)**. Furthermore, both diffusion-weighted images and apparent diffusion coefficient (5) images do not indicate any restrictions in diffusion **(D, E)**.

The total score of the scale for the assessment and rating of ataxia was 24, and the single-item scores were gait (score 5), stance (4), sitting (2), speech (2), finger chase (2), nose–finger test (2), fast alternating hand movement (3), and heel–shin slide (4). The patient's Mini-Mental State Examination score was within the normal range, and she failed to complete the Montreal Cognitive Scale due to tremors of the hands and head. The results for tumor markers, ceruloplasmin, immune-related autoantibodies such as the five items of thyroid function (thyroid stimulating hormone, free T3, free T4, thyroglobulin antibody and thyroid peroxidase antibody), three items of rheumatism (rheumatoid factors, anti-streptolysin O and high-sensitivity C-reactive protein), five items of immunity (immunoglobulin G, immunoglobulin A, immunoglobulin M, complement C3 and complement C4), antinuclear antibody, anticardiolipin antibody, and screening and confirmation of anti-neutrophil cytoplasmic antibody were not significantly abnormal. Lumbar puncture results for the patient showed no abnormalities. Brain MRI revealed speckled and small patchy abnormal signals bilaterally in the corona radiata, the paraventricular and frontal lobes, and the left temporal lobe. The lesions appeared hypointense on T1 images and hyperintense on T2 and FLAIR images. There were no obvious abnormal signals on diffusion-weighted images (DWI) and apparent diffusion coefficient maps (Figures 1A–E). The radiological diagnosis was multiple white matter demyelinating lesions. Color Doppler ultrasonography of the gynecological, abdominal, cardiac, carotid, and intracranial arteries revealed no obvious abnormalities.

**Figure 2:**
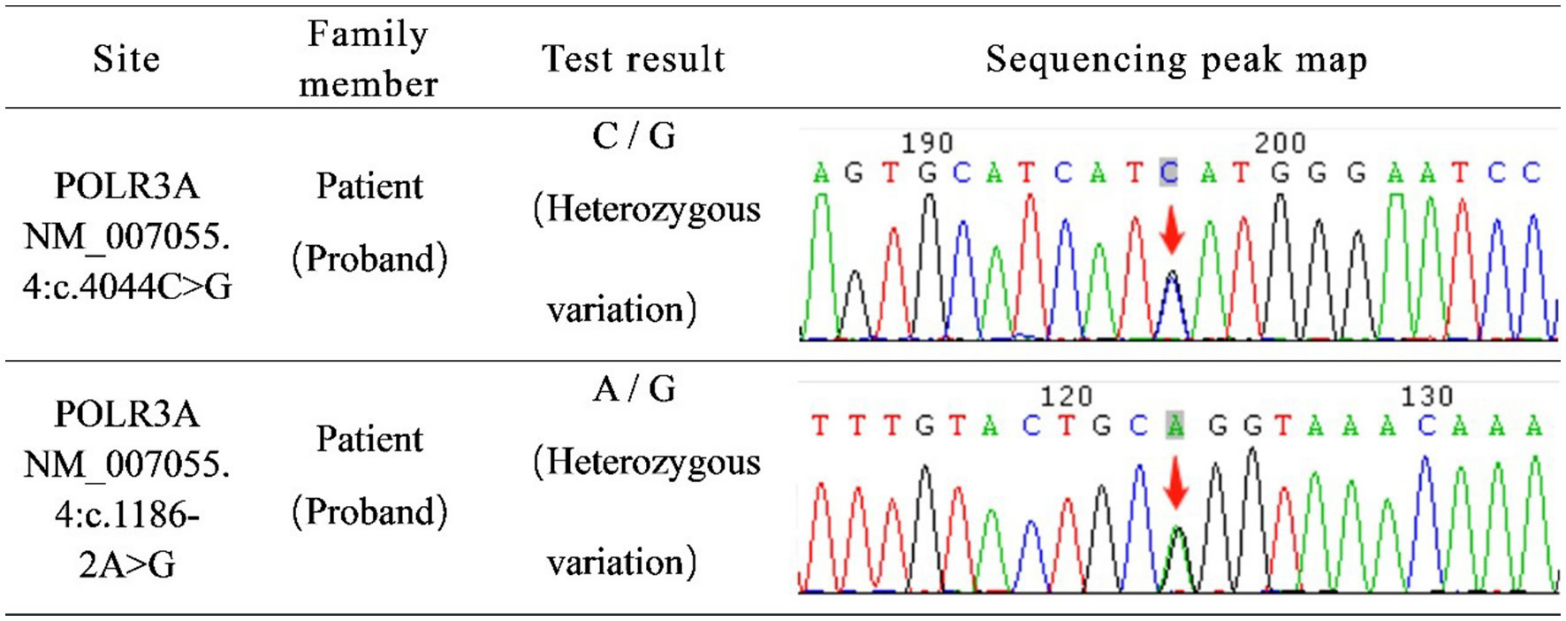
Genetic disease gene detection report: c.4044C > G and c.1186-2A > G of *POLR3A* gene were mutated.

The detection of hereditary disease genes revealed mutations, namely c.4044C > G and c.1186-2A > G, in *POLR3A* (Figure 2). According to the American College of Medical Genetics and Genomics (ACMG) guidelines, the genetic variant c.4044C > G is classified as PM2; however, its clinical significance remains unclear. The genetic variant c.1186-2A > G, classified as PVS1 and PM2, has the potential to influence RNA splicing, subsequently impacting protein function and causing disease. Respecting the patient's opinion, we did not perform genetic testing for her parents or son. Based on the results of physical examination, laboratory tests, imaging tests and gene reports, her condition was diagnosed as POLR3A-related leukodystrophy (HLD7).

We administered oral vitamin B12 and injectable acetylglutamine to nourish the nerves. No other drugs were used during the hospital stay. However, the symptoms did not show significant improvement during the 1 month of follow-up and appeared aggravated. The patient required assistance to walk and was unable to hold objects in her hands. After discharge, the patient was treated with vitamin B12, vitamin E, coenzyme Q, and traditional Chinese medicine, with Ganoderma lucidum, ginseng, and Poria Cocos as the main ingredients. As of recent follow-up (1 year after she was discharged), she is able to walk slowly alone and climb stairs, and the degree of head and hand tremors has reduced significantly. She is also able to hold objects and cook independently. However, her speech is slower and more indistinct than before.

## 3. Discussion

Leukodystrophies are a group of rare and refractory genetic diseases characterized by white matter abnormalities in the CNS, with a reported incidence of 1 in 7,500 live births (6). Onset may occur at any age, from prenatal life to senescence. Studies have shown that autosomal recessive mutations in *POLR3A* account for 3% of the unclassified autosomal recessive inherited and sporadic cases (7); therefore, early diagnosis and classification are essential. The clinical features of leukodystrophy caused by *POLR3A* mutations include neurological, dental, ophthalmic, and endocrine changes, with at least one of these criteria noted in each patient. However, the endocrine symptoms and developmental abnormalities associated with this disease have not been thoroughly studied (3). Neurological signs and symptoms include cognitive impairment and cerebellar symptoms such as ataxia and nystagmus. More than 80% of patients have cerebellar atrophy, while only a few have extrapyramidal signs such as dystonia and tremors (8). At the time of onset, the only manifestation may be simple cognitive impairment, as motor symptoms may not be present. This disease often exhibits non-neural manifestations, including dental problems such as tooth loss, endocrine problems such as hypogonadism reduction, and ophthalmic problems such as optic atrophy and myopia (9). Brain MRI often shows diffuse hypomyelination in the whole-brain white matter, which can accumulate in the internal capsule, external capsule, deep white matter, and corpus callosum. Subcortical U fibers are often not involved, and cerebellar atrophy and superior cerebellar peduncle hyperintensity are occasionally seen (10). In studies published in the last 5 years (8, 11–26), we collected data from cases of myelin dystrophic leukodystrophy caused by *POLR3A* mutation and summarized the relevant characteristics in Table 1.

**Table 1:** The table shows the cases of leukodystrophy caused by *POLR3A* mutation published in recent years, and summarizes the relevant characteristics.

**P**	**Sex**	**Age (years)**	**Age of onset (years)**	**Brain magnetic resonance imaging (MRI)**	**The site of the Polr3A mutation**	**Neurological symptoms**	**Non-neurological symptoms**
1	F	42	15	Brain MRI described whole brain cortical atrophy on T1-weighted images and diffuse white matter hypomyelination in the bilateral cerebral hemispheres on T2-weighted images and fluid attenuated inversion recovery (FLAIR).	c.1911 + 18C > T	Ataxia and cognitive impairment	Hypodontia, amenorrhea, and short stature
2	F	1.5	5 months	Brain MRI showed diffuse signal alterations in the entire cerebral white matter with involvement of the internal capsule, external capsule, frontal deep white matter and corpus callosum appearing hyperintense on T2 weighted and hypointense on T1 weighted images consistent with hypomyelination.	chr10: 79760789; C > T; c.2423G > A	Horizontal, nystagmus, hypertonia and limb spasticity	Facial dysmorphism, hypodontia, lush body hair, and clitoris hypertrophy
3	M	39	30+	On T2-weighted images (upper parts), there is a diffuse and symmetric hyperintensity of the supratentorial WM, involving also the subcortical U fibers, posterior limbs of internal capsules and external capsules. On T1 weighted images (lower parts), WM signal is mildly hypointense in some areas.	c.3820A > G, p.Thr1274Pro; c.3511_3595-58del	Cognitive impairment	Sexual dysfunction
4	M	46	30+	On T2-weighted images (upper parts), there is a diffuse and symmetric hyperintensity of the supratentorial WM, involving also the subcortical U fibers, posterior limbs of internal capsules and external capsules. On T1-weighted images (lower parts), WM signal is uniformly normal.	c.2554A > G (p.Met852Val)	Limb tremor, limb spasm, and cognitive impairment	-
5	F	41	1	Brain MRI T2-weighted images showed brain atrophy and diffuse hyperintensity in the white matter, indicating hypomyelination. In contrast, myelination was relatively well-preserved in the globus pallidus, cerebellar dentate nucleus, and anterior lateral nucleus of the thalamus. Thinning of the corpus callosum and brain stem and cerebellar atrophy were also profound.	C.2554A > G (p.M852V); C.2668G > T (p.V890F)	Ataxia and limb spasticity	Hypodontia, amenorrhea, and pubic hair hypoplasia of the breast
6	M	38	25	Brain MRI demonstrated marked cerebellar atrophy and bilateral symmetrical T2 hyperintense lesions with preserved U fibers.	c.G1745: p.R582P; NM_007055	Ataxia, nystagmus and limb spasticity	-
7	F	31	-	Diffuse hypomyelination of the subcortical and deep white matter, enlarged high convexity CSF spaces, ponto-cerebellar hypoplasia, and thin corpus callosum.	c.3718G > A; c.1795C > A	-	-
8	M	23	18 months	Slight peri- and supra-ventricular white matter hyperintensities and cortical atrophy.	c.1795C > A; c.328A > G	Cognitive impairment	Cataract
9	M	9	-	Hypomyelination and abnormal signal in brain stem.	c.661_662insCCT; c.1770 + 5 G > C	Ataxia, nystagmus and cognitive impairment	-
10	F	18	-	Brain MRI showed bilateral cerebral hemisphere myelin dysplasia, brain atrophy, thin corpus callosum, and small pituitary gland with uneven reinforcement and enlarged ventricles.	c.3013C > T; c.1757C > T	Ataxia and cognitive impairment	-
11	F	51	35	MRI brain demonstrated global atrophy and T2-FLAIR white matter changes, sparing subcortical structures, with U-fiber involvement.	NM_007055.3:(c.367_369delA AG) (p.Lys123del); NM_007055.3:(c.2554A > G) (p.Met852Val)	Limb tremor and cognitive impairment	Hyperdontia
12	M	30	16	Cerebellar atrophy	c.1909 + 22G > A; c.1031G > T (p.Arg344Leu)	Dysarthria, intentional tremor and gait ataxia	Myopia
13	F	59	49	Cerebellar atrophy, thin corpus callosum, superior cerebellar peduncle hyperintensity and corticospinal tract.	c.1909 + 22G > A; c.2788-2A > T	-	-
14	M	54	30	Superior cerebellar peduncle hyperintensity and corticospinal tract.	c.1909 + 22G > A; c.2394 T > A (p.Cys798^*^)	Gait ataxia	-
15	M	35	13	Superior cerebellar peduncle hyperintensity and corticospinal tract.	c.1909 + 22G > A; deletion of exons 14–18	Dysarthria, intentional tremor, gait ataxia and seizures	Subclinical hypogonadism (low FSH/LH)
16	F	38	29	Cerebellar atrophy	c.1909 + 22G > A; c.3201_3202delGC (p.Arg1069fs^*^2)	Dysarthria	Hypodontia
17	F	64	13	Superior cerebellar peduncle hyperintensity and hypomyelination.	c.1909 + 22G > A; c.4073G > A (p.Gly1358Glu)	Dysarthria, intentional tremor and gait ataxia	-
18	M	58	15	Superior cerebellar peduncle hyperintensity	c.1909 + 22G > A; c. 4073G > A (p.Gly1358Glu)	Dysarthria, intentional tremor and gait ataxia	-
19	F	47	36	Thin corpus callosum and superior cerebellar peduncle hyperintensity.	c.1909 + 22G > A; c.3733C > T (p.Arg1245^*^)	Dysarthria and intentional tremor	-
20	M	41	22	Cerebellar atrophy, thin corpus callosum and superior cerebellar peduncle hyperintensity.	c.1909 + 22G > A; c.3733C > T (p.Arg1245^*^)	Dysarthria	-
21	F	32	19	Cerebellar atrophy	c.1909 + 22G > A; c.2554A > G (p.Met852Val)	Intentional tremor and gait ataxia	-
22	M	75	19	Brain MRI showed neither white matter abnormality nor cerebellar atrophy.	c.1909 + 22G > A (p.Y637Cfs^*^14);c.1051C > T (p.Arg351^*^)	Gait ataxia, nystagmus and dysarthria	-
23	M	46	41	white and gray matter atrophy	NM_007055: c.(2554A > G); NP_008986: p.(Met852-Val)	Gait ataxia and cognitive impairment	-
24	F	34	19	Thin corpus callosum and the cortical/subcortical atrophy which included the brainstem and cerebellum including cerebellar peduncles.	NM_007055: c.(2325C > G); NP_008986: p.(Asn775Lys)	Ataxia, nystagmus and cognitive impairment	Bilateral dysplasia of the hip and primary amenorrhea
25	F	-	9 months	Brain MRI showed the extra cerebral space widening at six months old and frontotemporal space widening, delayed myelination or hypomyelination of white. Matter in the focal area around the posterior horn of the bilateral lateral ventricles.	c.1771-6C > G; c.2611del (p.M871Cfs^*^8),	Cognitive impairment, nystagmus, moderate dysarthria and motor decline	Short stature, hypodontia, delayed dentition and prominent body hair
26	M	18+	6 months	Brain MRI revealed bilateral symmetric atrophy and increased signal of the caudate nucleus and the putamen. The white matter and red nucleus showed no abnormal signal changes, and cerebellum was normal.	c.1771-6C > G p. (Pro591Metfs^*^9); c.791C > T p. (Pro264Leu)	Cognitive impairment, dysarthria and gait ataxia	Growth delay
27	F	23	6 months	Brain MRI showed bilateral symmetric atrophy and increased signal of the striatum and cerebral atrophy.	c.1771-6C > G p. (Pro591Metfs^*^10); c.2671C > T p.(Pro264Leu)	Cognitive impairment, dysarthria, dystonia and ophthalmoparesis	Hypodontia
28	F	15 (died)	1	Brain MRI showed bilateral-atrophy-associated increased signal in the striatum and frontal predominant cerebral atrophy. Neither signal changes in red nucleus nor cerebellar atrophy were recognized.	c.1771-6C > G p. (Pro591Metfs^*^11); c.2671C > T p. (Pro264Leu)	Cognitive impairment, dysphagia and dystonia	-
29	F	37	At birth	Brain MRI revealed cortical lesions of the angular and fusiform gyri, likely consequences of traumatic brain injuries, increased T2 signal intensity in the dorsal spinal cord, as well as corticospinal tract and cerebellar peduncles hyperintensities on MRI-fluid attenuated inversion recovery (FLAIR)	c.3336G > A	Bilateral hearing loss, aphonia, spastic quadriplegia	Facial deformity, postnatal growth retardation, global lipodystrophy, joint contractures, thoracic hypoplasia, scoliosis, anodontia, hypogonadotropic hypogonadism
30	F	16	12	Brain MRI revealed pathological hyperintensity on T2-weighted images secondary to hypomyelination was seen in periventricular white matter and centrum semiovale. Mild atrophy of the cerebrum, cerebellum, and corpus callosum was also detected.	c.2005C > G (p.R669G) (p.Arg669Gly)	Cognitive impairment	Hypogonadotropic hypogonodism
31	M	6	18 months	Brain MRI revealed abnormal high signal changes in bilateral caudate and putamen (striatum).	c.1771-6C > G; c.4037G > A (p.C1346Y)	Cognitive impairment, seizures, dysphagia	Developmental retardation

P, patient; F, female; M, male; -,unknown or nothing.

The two pathogenic genes that encode the largest subunit of human RNA polymerase III (Pol III) are *POLR3A* and *POLR3B*. Mutations in these two genes can result in four overlapping phenotypes of hypomyelinating leukodystrophy, which can be divided into the following syndromes according to clinical features: hypomyelination, hypodontia, and hypogonadotropic hypogonadism (4H syndrome); ataxia, delayed dentition, and hypomyelination (ADDH); tremor-ataxia with central hypomyelination (TACH); leukodystrophy with oligodontia (LO); and hypomyelination with cerebellar atrophy and hypoplasia of the corpus callosum (HCAHC) (27). It has been shown that in almost all patients with *POLR3A* and *POLR3B* related leukodystrophy, myelination of the optic radiation is always accompanied by hypomyelination and that the ventrolateral thalamus and dentate nucleus have relatively low signal intensity on T2. Supratentorial atrophy is common in adult patients and rare in children younger than 10 years (28). Reports also state that different genes cause some differences in brain MRI. Patients with *POLR3B* gene mutations have smaller cerebellums, larger fissures, thinner cortices, and decreased basal white matter, suggesting cerebellar atrophy. MR images of patients with *POLR3A* mutations show significantly lighter changes in the cerebellar hemispheres and cerebellar vermis, indicating only a slight myelin sheath reduction in the centrum semiovale and cerebellar white matter (29). The symptoms and prognoses of the two patients were also different. Patients with *POLR3A* mutations progress faster and have a shorter life expectancy than those with *POLR3B* mutations. It is worth noting that in patients with the *POLR3A* mutation, the disease onset is slightly later than in patients with other mutations. Compared with patients with *POLR3B* mutations, most patients with *POLR3A* mutations achieve independent walking (28).

In this case, the patient had the non-neurological symptoms of tooth loss. Based on the results of genetic testing, the patient was diagnosed with leukodystrophy type 7 and further classified as having LO syndrome based on the MRI report and the presence of cerebellar ataxia and nystagmus. For this patient, we used aceglutamide which is the acetyl compound of glutamine, which passes the blood-cerebrospinal fluid barrier and decomposes into glutamic acid and gamma-aminobutyric acid (GABA). Glutamate is involved in information transmission in the central nervous system. Gamma-aminobutyric acid can antagonize the excitability of glutamate, improve nerve cell metabolism, reduce oxygen consumption and reduce the effect of blood ammonia, and improve brain function (30). However, this drug was not effective in this patient. To date, no effective treatment has been found for this disease; however, symptomatic treatment can still be useful in improving muscle tone disorders and swallowing dysfunction, thereby further improving the quality of life of patients. For the treatment of dystonia, the first-line drugs we can use are anticholinergic drugs, such as trihexyphenidyl; the second-line drugs are reversible dopamine depleting agents, such as tetrabenazine; other drugs such as baclofen, benzodiazepines, levodopa, botulinum toxin, etc. For refractory patients, we can try deep brain stimulation (31). It has been reported that a patient with POLR3A associated leukodystrophy and Parkinson's disease had complete remission of bradykinesia and left leg tremor after deep brain stimulation. However, there is no systematic data to confirm the effectiveness of deep brain stimulation in patients with POLR3A associated leukodystrophy (18). Notably, ibuprofen may be used for the treatment of HLD 7; however, its efficacy and mechanism require further exploration (4).

## 4. Conclusion

As leukodystrophy caused by the *POLR3A* mutation is relatively rare, reports in this area are minimal. Although MRI features are helpful for diagnosis in patients lacking basic non-neurological features, the disease remains difficult to diagnose because it is not well understood. Therefore, we report and analyse a case of leukodystrophy caused by a missense mutation in *POLR3A*. Leukodystrophy caused by the *POLR3A* mutation often has a poor prognosis, affecting the life span of patients; thus far, there is no cure for the disease except for some symptomatic treatments, such as reducing muscle tension. We still need to further explore and study new treatment methods to improve the quality of life of patients while increasing their life expectancy, which is an important work in the future.

## Data availability statement

The original contributions presented in the study are included in the article/supplementary material, further inquiries can be directed to the corresponding author.

## Ethics statement

The studies involving humans were approved by Clinical Research Management Committee of the First Hospital of Jilin University. The studies were conducted in accordance with the local legislation and institutional requirements. The authors declare that all the medical records in this study are from previous diagnoses and treatments. The subject's privacy and personally identifiable information are protected. Written informed consent was obtained from the individual for the publication of any potentially identifiable images or data included in this article.

## Author contributions

LS: Investigation, Writing–original draft. SH: Funding acquisition, Writing—review & editing. WL: Conceptualization, Writing—review & editing. HM: Writing—review & editing. WZ: Writing—review & editing.
